# The offspring sex ratio at birth in one of the largest human harems

**DOI:** 10.17179/excli2024-7020

**Published:** 2024-04-29

**Authors:** Mostafa Saadat

**Affiliations:** 1 Department of Biology, School of Science, Shiraz University

## ⁯

The sex ratio at birth has been extensively studied in humans and other mammals. Evidence suggests that changes in sex ratio (male proportion) are a reflection of stresses introduced into a family or population (Grech, 2018[9]). These stresses can include economic stress (Song, 2014[23]), sudden natural events such as earthquakes (Fukuda et al., 1998[8]; Saadat, 2008[18]), pandemics (Saadat, 2021[19]), the outbreak of war (Ansari-Lari and Saadat, 2002[1]), and psychological stressors (Saadat and Ansari-Lari, 2004[20]). Multiple lines of evidence suggest that sex ratio at birth is an important indicator for monitoring the health of human populations (Grech, 2018[9]).

Although almost all human families consist of one woman and one man, a small number of families may practice polygamy or polyandry. While polyandry is no longer widely practiced in most human societies, polygamy still persists. It is important to note that the majority of polygamous families typically involve one man living with his two or three wives, although there are exceptions where one man has a large number of wives. Typically, men who practice polygamy have a higher economic status than societal norms.

Fath-Ali Shāh (1769-1834) served as the second king of the Qājār dynasty (r. 1789-1797), ruling from 1797 until October 1834, when he passed away. It is worth noting that his family structure, especially his harem, is intriguing. European travelogues, as well as Iranian literature of the king's time and historical accounts written a few decades after his death, indicate that he had a very large harem. He is famous among Iranians for his legendary harem. Sources indicate that the king had a large number of children and grandchildren. "Itezad ol-Saltaneh”, the son of the king, wrote a history book, "Oksir ol-tavarikh," in which he mentioned that from the creation of Adam to the present day, no one has ever had such an abundance of offspring as Fath-Ali Shāh (Itezad ol-Saltaneh, 1991: p.183[11]). Another historian (Khavari, 2001, vol 2: p. 969[13]) also claimed that no historian had ever mentioned a king with such a large number of children from different ethnic groups. The number of wives of Fath-Ali Shāh was reported to be between 300 and 1000 (Sepehr, 1998, vol 1: p. 551[21]; Azod ol-Dowleh, 1997: p.194[3]). Sepehr (1998, vol 1: pp. 551-562[21]) recoded 157 Fath-Ali Shāh's wives.

Fath-Ali Shāh had many children and grandchildren. In his travelogue, James Justinian Morier (1782-1849) reported that “It sometimes happens that many of his wives are delivered on the same night!” (Morier, 1812: p. 226[16]). According to historian Upton (1960: p. 4[26]), he "is famous among Iranians for three things: his exceptionally long beard, his wasp-like waist, and his progeny". It should be noted that the increase in the number of his wives made it necessary to create a space where his spouses could live together with their children and servants. Such spaces were called harems. The size of the harem depended on the financial power and social status of its owner. Although some Qajar monarchs had harems, the number of wives they had was not equal to that of Fath-Ali Shāh. Men were not allowed to enter the harem, and all matters related to the harem were handled by women. Only the king, his sons, and prepubescent male children who lived with their mothers were allowed to enter the harem. In addition, males who had been castrated before puberty were also allowed entry. Morier (1812: pp. 225-226[16]) and Taj ol-Saltaneh (1999: pp. 13-26[24]) have provided interesting information about the harem and its rules. 

There have been some studies of animal harems and their effect(s) on pregnancy outcomes, including the sex ratio of offspring at birth and the relationship between females living in a given harem. In contrast, there is a lack of similar studies in the human population. Therefore, the present historical study was conducted.

The study examines the sex ratio of children born in the harem of Fath-Ali Shāh in comparison to that of the general Iranian society two centuries earlier. The main problem of the current study is related to obtaining raw data. Fortunately, there are some reports in historical books about the number of children of Fath-Ali Shāh. Here I have used four first-hand sources in the history of the Qājār dynasty. They are described below:

1. “Oksir ol-tavarikh” was written by “Itezad ol-Saltaneh” in 1843[11].

2. “Tārix-e Zol-Qarneyn” was written by “Mirza Fazlollah Shirazi, Khavari” in 1846[13].

3. “Nasikh ol-Tawārikh” was written by “Sepehr” in 1854[21].

4. “Tārikh-e Azodi” was written by “Azod ol-Dowleh” in 1887[3].

The sources do not give an exact number of the king's wives and offspring (Sepehr 1998, vol 1: p. 551[21]; Azod ol-Dowleh, 1997: p. 194[3]; Khavari, 2001, vol 2: pp. 969-970, 1059[13]; Itezad ol-Saltaneh, 1991: p. 183[11]). They do, however, consistently mention a large number of children, especially male children. According to James Justinian Morier (1812: p. 226[16]), “The King's family consists of sixty-five sons. As they make no account of females, it is not known how many daughters he may have”.

Khavari (2001, vol 2: pp. 969-970, 1059[13]) and Sepehr (1998, vol 1: pp. 521-551[21]) have reported more data about offspring of Fath-Ali Shāh in comparison to other sources and have reported very similar data in comparison to each other. The historians' method of data collection is unclear; whether they used a single source or collected data independently. They have reported that the king had a total of 260 offspring, of which 101 were living at the time of his death. Here I have accepted the number of children as 260 for the present study. It should be noted that the majority of the king's children (159) died during his lifetime. However, they do not specify the sex of these children. This means that the mortality rate among the king's descendants was about 61.2 % (= 159 × 100/260). Jakob Eduard Polak (1818-1891), an Austrian physician, was the personal physician of the Qājār king, Nāṣer-od-Dīn Shāh (r. 1848-1896). He spent nine years in Iran and played an important role in introducing modern medicine to Iran (Azizi, 2005[2]). Polak published his Experiences in Iran, which is one of the outstanding ethnographic works on the Iranian population in the 19^th^ century. In chapter 6 of his book, he recorded valuable information about diseases, fertility, and mortality in Iranian society at that time. He said that despite the high birth rate, the population did not increase because of the high infant and child mortality rates. He pointed out that due to the high mortality rate, out of 6 children, 2 or less than 2 children may remain for the family (Polak, 1865: pp. 216-217[17]). This means that the infant and child mortality rate was about 66.7 %. Therefore, the recorded high mortality rate among the king's descendants reported by Sepehr and Khavari is very similar to Polak's report.

Unfortunately, mortality data differentiated by sex from two centuries ago in Iran is unavailable. However, according to statistical reports by Sepehr (1998, vol. 1: p. 521[21]), 71.4 % of the deceased were male. To estimate the number of sons and daughters of Fath-Ali Shāh, we made two assumptions: 1) the ratio of deceased males to deceased offspring of the king was 0.714, and 2) the ratio of deceased males to deceased offspring of the king was 0.600. The second assumption is more conservative than the first. The king had a total of 159 deceased children, according to two assumptions: 113 sons and 46 daughters under the first assumption, and 95 sons and 64 daughters under the second assumption. Sepehr (1998, vol 1: p. 521[21]) and Khavari reported that the king had 55 surviving sons and 46 surviving daughters during his demise. Hence, the king had a total of 168 boys and 92 girls under the first assumption and 150 boys and 110 girls under the second assumption. Taken together, it is estimated that 0.6462 and 0.5769 of Fath-Ali Shāh's progeny were male under the first and second assumptions, respectively.

To evaluate the sex ratio at birth of the king's offspring to that of the general population, it is necessary to estimate the general population's sex ratio during that time period. Unfortunately, statistical data is unavailable for that period. Two assumptions were made to estimate the sex ratio of the general population. The first assumption is that the sex ratio at birth of Fath-Ali Shāh's grandchildren (0.5007) corresponds with that of society, based on reports of Sepehr (1998, vol 1: p. 521[21]) and Khavari (2001, vol 2: p. 1059[13]). The second assumption is more conservative, estimating the sex ratio at birth for society to be 0.5100. Given the above two assumptions for estimating offspring sex and two for the baseline sex ratio at birth, four statistical comparisons should be made (Supplementary information, Table S1). The statistical analysis revealed a significant male bias among Fath-Ali Shāh's offspring compared to the general population under four combinations of assumptions. The second set of assumptions was more conservative than the first set, resulting in a very conservative combined assumption. Interestingly, there was a statistically significant difference between the observed and expected values (χ^2 ^= 4.65, df = 1, p = 0.031). It should be noted that there was a gender preference in the Iranian population. However, historical sources do not support the assumption that female new-borns were killed.

To the best of my knowledge, there has been no study of the sex ratio at birth within large human harems. This is due to the rarity of such family structures, both past and present. However, Guinness World Records notes that by the year 1703, Moulay Ismail, the last Sharif emperor of Morocco (1672-1727), had a total of 525 sons and 342 daughters. French diplomat Dominique Busnot, who visited Morocco during Moulay Ismail's reign, reported these figures. I am not familiar with the history of Morocco in the 18^th^ century, so I cannot make any assertions on the data's accuracy. Nevertheless, it appears that the sex ratio of Moulay Ismail's offspring is significantly high, as is that of Fath-Ali Shāh's children, thus confirming the current finding.

One study reported that billionaires tended to have more sons than the average population, and when the billionaires were categorized by gender, women who married billionaires had more male offspring than the general population and female billionaires themselves. However, the offspring sex ratio of female billionaires did not differ from that of the general population (Cameron and Dalerum, 2009[4]). According to the Trivers-Willard hypothesis, mothers with different levels of resources in human populations would benefit differently from producing sons or daughters. Specifically, mothers with more resources would benefit from producing sons, while those with fewer resources would benefit from producing daughters (Trivers and Willard, 1973[25]). As noted in the introduction, Fath-Ali Shāh had several hundred wives residing in his harem. Since the king was the owner of the harem, the inhabitants of the harem had no economic problems. Therefore, the gender imbalance in Fath-Ali Shāh's descendants in favor of male offspring is consistent with the Trivers-Willard hypothesis and is comparable to male billionaires marrying non-billionaire women. 

One can imagine that if Fath-Ali Shāh were not the king, he would be one of the men who cheated on his wife. By forming a large harem and collecting several hundred wives, he was able to respond positively to his variety of sexual desires. It has been reported that testosterone levels are higher in men who cheat on their wives (Edelstein et al., 2011[6]; Klimas et al., 2019[14]). In response to sexual stimuli, male testosterone levels increase significantly (Escasa et al., 2011[7]). Testosterone treatment was reported to significantly increase sexual activity, sexual desire and erectile function in older men compared to placebo using data from the double-blind Sexual Function Trial (Snyder et al., 2018[22]). A positive correlation between serum testosterone levels and orgasm frequency has been reported (Knussmann et al., 1986[15]). On the other hand, according to the James hypothesis, an increase in testosterone at the time of conception may increase the sex ratio in favor of the male sex (James, 2015[12]). Taken together, it can be suggested that Fath-Ali Shāh's testosterone level was higher than that of the population, and on the other hand, various sexual stimulants in his harem increased the hormone level and subsequently his sexual activity. It is possible that the increase in the sex ratio of Fath-Ali Shāh's offspring was related to his testosterone level.

Taj ol-Saltaneh (1884-1936) was the daughter of one of the Qājār kings and had experience living in her father's harem. She reported valuable information from inside the harem (Taj ol-Saltaneh, 1999: pp. 13-26[24]). It should be noted that her father's harem was much smaller than Fath-Ali Shāh's harem. If we consider the total count of wives and daughters of Fath-Ali Shāh along with their accompanying servants, dancers, and singers, the number of women could exceed three thousand. They lived in a relatively small space. As I mentioned earlier, since the king was the owner of the harem, the inhabitants of the harem did not feel any economic problems, but the living together of a large number of women had created special conditions. Living with a large number of women in a harem, especially women who share the same husband, creates rivalry among them.

A study of a wild population of Chacma baboons in the Tsaobis Leopard Park in central Namibia shows that aggression between females is a function of the number of swollen females in a group. The frequency of aggression exchanged between females increases with the number of sexually receptive females in the group (Huchard and Cowlishaw, 2011[10]). It is reasonable to assume that in human populations, females with a common husband, which occurs in large harems, experience aggression among themselves and are involved in intense competition for their husband's sexual attention. Taj ol-Saltaneh (1999: pp. 31-32[24]) reports and explains some types of aggressive behavior and competition among the king's wives in the harem.

In one study, two breeding systems, namely the temporary monogamous system and the harem system, were used to investigate the effect of breeding system on the sex ratio at birth in Syrian hamsters (*Mesocricetus auratus*). In this experiment, both groups of animals were kept under the same environmental conditions, and the body condition of the dams and pups was similar in both groups. Interestingly, the researchers found a male-biased sex ratio in the harem system litter (Chelini et al., 2011[5]), which is in good agreement with the present finding.

The sex ratio at birth in large harems may differ from that in small human harems. Generalizing the current findings to small harems requires additional information and further research. Finally, the use of historical records for analysis is the primary limitation of this study.

## Conflict of interest

None.

## Supplementary Material

Supplementary information

## References

[1] Ansari-Lari M, Saadat M (2002). Changing sex ratio in Iran, 1976-2000. J Epidemiol Communy Health.

[2] Azizi MH (2005). Dr. Jacob Eduard Polak (1818-1891): The pioneer of modern medicine in Iran. Arch Iran Med.

[3] Azod ol-Dowleh MAK, Navaei H-M (1997). Tarix-e Azodi.

[4] Cameron EZ, Dalerum F (2009). A Trivers-Willard effect in contemporary humans: male-biased sex ratios among billionaires. PLoS One.

[5] Chelini MO, Souza NL, Otta E (2011). Breeding system can affect the offspring sex ratio in Syrian hamsters. Lab Anim.

[6] Edelstein RS, Chopik WJ, Kean EL (2011). Sociosexuality moderates the association between testosterone and relationship status in men and women. Horm Behav.

[7] Escasa MJ, Casey JF, Gray PB (2011). Salivary testosterone levels in men at a U.S. sex club. Arch Sex Behav.

[8] Fukuda M, Fukuda K, Shimizu T, Møller H (1998). Decline in sex ratio at birth after Kobe earthquake. Hum Reprod.

[9] Grech V (2018). Correlation of sex ratio at birth with health and socioeconomic indicators. Early Hum Dev.

[10] Huchard E, Cowlishaw G (2011). Female-female aggression around mating: an extra cost of sociality in a multimale primate society. Behav Ecol.

[11] Itezad ol-Saltaneh AQM, Kianfar J (1991). Oksir ol-tavarikh.

[12] James WH (2015). Proximate causes of the variation of the human sex ratio at birth. Early Hum Dev.

[13] Khavari (pen name for Mirza Fazlollah Shirazi), Afsharfar N (2001). Tarix-e Zol-Qarneyn.

[14] Klimas C, Ehlert U, Lacker TJ, Waldvogel P, Walther A (2019). Higher testosterone levels are associated with unfaithful behavior in men. Biol Psychol.

[15] Knussmann R, Christiansen K, Couwenbergs C (1986). Relations between sex hormone levels and sexual behavior in men. Arch Sex Behav.

[16] Morier JJ (1812). A journey through Persia, Armenia, and Asia Minor, to Constantinople, in the Years 1808 and 1809.

[17] Polak JE (1865). Persien: das Land und seine Bewohner. Ethnographische Schilderungen.

[18] Saadat M (2008). Decline in sex ratio at birth after Bam (Kerman Province, Southern Iran) earthquake. J Biosoc Sci.

[19] Saadat M (2021). Sex ratio at birth in COVID-19 era: A report from Iran. EXCLI J.

[20] Saadat M, Ansari-Lari M (2004). Sex ratio of birth during wartime and psychological tensions. Hum Reprod.

[21] Sepehr, Mohammad-Taqi Lesan ol-Molk, Kianfar J (1998). Nasikh ol-Tawarikh.

[22] Snyder PJ, Bhasin S, Cunningham GR, Matsumoto AM, Stephens-Shields AJ, Cauley JA (2018). Lessons from the testosterone trials. Endocr Rev.

[23] Song S (2014). Malnutrition, sex ratio, and selection: a study based on the great leap forward famine. Hum Nat.

[24] Taj ol-Saltaneh, Erfanian M (1999). Khaterate Taj-o-Saltaneh.

[25] Trivers RL, Willard D (1973). Natural selection of parental ability to vary the sex ratio of offspring. Science.

[26] Upton JM (1960). The history of modern Iran: an interpretation.

