# Supplementary material for: The offspring sex ratio at birth in one of the largest human harems
Source: EXCLI J. 2024 Apr 29;23:624–8. doi: 10.17179/excli2024-7020 (PMC11180934; doi:10.17179/excli2024-7020)
Supplement: Supplementary information [file EXCLI-23-624-s-001.pdf]

EXCLI Journal 2024;23:624-628 – ISSN 1611-2156
Received: January 24, 2024, accepted: April 26, 2024, published: April 29, 2024

Supplementary information to:

Letter to the editor:

THE OFFSPRING SEX RATIO AT BIRTH IN ONE OF THE LARGEST HUMAN HAREMS

Mostafa Saadat

Department of Biology, School of Science, Shiraz University, Shiraz 71467-13565, Iran, Tel: +98-71-36137432; Fax: +98-71-32280926; E-mail: saadat@shirazu.ac.ir

https://dx.doi.org/10.17179/excli2024-7020

This is an Open Access article distributed under the terms of the Creative Commons Attribution License (http://creativecommons.org/licenses/by/4.0/).

Supplementary Table 1: Sex ratio at birth in the descendants of Fath-Ali Shāh Qājār, Iran

Gender of Fath-Ali Shāh's offspring using two assumptions for sex of dead offspring			Results of comparisons under two assumptions for base line of sex ratio at birth	
**Assumptions**	**Males**	**Females**	**Assumption I**	**Assumption II**
**I**	168	92	χ^2=22.18, p<0.0001	χ^2=22.15, p<0.0001
**II**	150	110	χ^2=6.14, p=0.013	χ^2=4.65, p=0.031

Note: df=1 for all comparisons. Two assumptions I and II for estimating the number of sons and daughters of Fath-Ali Shāh Qājār, as well as two assumptions about the sex ratio in the general population, are given on page 626 of the text of the letter.
